# Effects of Ferulic Acid on Lipopolysaccharide-Induced Oxidative Stress and Gut Microbiota Imbalance in Linwu Ducks

**DOI:** 10.3390/antiox13101190

**Published:** 2024-09-30

**Authors:** Yang Liu, Xuan Huang, Chuang Li, Ping Deng, Xu Zhang, Yan Hu, Qiuzhong Dai

**Affiliations:** Hunan Institute of Animal Husbandry and Veterinary Medicine, Changsha 410131, China; yangl035@163.com (Y.L.); huangxuan1@hotmail.com (X.H.); lichuang2@hotmail.com (C.L.); dengping2@hotmail.com (P.D.); zhangxu2024@hotmail.com (X.Z.); huyan13579@hotmail.com (Y.H.)

**Keywords:** metagenomics, metabolomics, intestinal microbiota, ferulic acid, Linwu duck, antioxidant, PPAR signaling pathway, phenylalanine metabolism

## Abstract

Oxidative stress is a major factor that limits the development of the poultry industry. Ferulic acid (FA) has an antioxidant effect in birds, but the mechanism is not fully understood. In this study, we stimulated oxidative stress in 28-day-old female Linwu ducks by lipopolysaccharide (LPS) and fed them a diet supplemented with FA for 28 days. Results showed that FA alleviated LPS-induced growth performance regression, oxidative stress, and microbiota imbalance in ducks. An integrated metagenomics and metabolomics analysis revealed that *s_Blautia_obeum*, *s_Faecalibacterium_prausnitzii*, *s_gemmiger_formicilis*, and *s_Ruminococcaceae_bacterium* could be the biomarkers in the antioxidant effect of FA, which interacted with dihydro-3-coumaric acid, L-phenylalanine, and 13(S)-HODE, and regulated the phenylalanine metabolism and PPAR signaling pathway. This study revealed the mechanism of the antioxidant effect of FA, which provided evidence of applying FA as a new antioxidant in commercial duck production.

## 1. Introduction

Oxidative stress is a major factor that lessens the economic income of the poultry industry. The main cause of oxidative damage is the overproduction of reactive oxidative species (ROS), which are generated from the intracellular energy metabolism in mitochondria and ubiquitous in cells [1]. Under normal physiological conditions, ROS are maintained at a balanced level by the antioxidant defense system, including antioxidant enzymes, free radical scavenging antioxidants, and damaged repair or removal molecules [2]. However, intensive farming density, low maintenance, and genetic selection for rapid growth cause metabolic disorders in birds that lead to excessive ROS generation [3,4,5]. Oxidative stress occurs once ROS concentration increases beyond the scavenging capacity of the antioxidant defense system. Oxidative stress jeopardizes the gastrointestinal function, growth performance, meat and egg quality, and fertility of birds [6,7]. As antibiotics became restricted in feed, it was urgent to develop safe and efficient feed additives to alleviate the influence of oxidative stress in the poultry industry.

As the main digestive and immune system in birds, the gastrointestinal tract, together with the gut microbiota, is influenced by oxidative stress. Extensive exposure to oxidative stress increases the gut permeability and disturbs microbial homeostasis. It allows pathogenic bacteria invasion, which induces intestinal malfunction and diminishes nutrition digestion and absorption [8]. On the other hand, gut microbiota and the intestine constitute the defense line against oxidative stress. The normal gut microbiota composition and complete intestinal mucosa layer determine the host’s antioxidant capacity. Furthermore, bacteria-synthesized metabolites, such as amino acids and vitamins, enhance the intestine function and host antioxidant capacity [9]. It is suggested that gut microbiota could be the potential regulators or participants in oxidative stress.

Ferulic acid (FA) is a phenolic acid with a resonance-stabilized phenoxy radical. It is the main active ingredient of the Chinese herbs Chuangxiong (*Ligusticum chuanxiong*) and Danggui (*Angelica sinensis*) and widely exists in plant cell walls [10]. FA has multiple bioactivities, such as anti-bacteria, antioxidant, anti-inflammatory, anti-obesity, anti-tumor, and anti-arteriosclerosis activities, which allows it to be widely used in food preservation, cosmetics production, and clinical medication [11,12]. A pharmacokinetic study showed an efficient absorption rate of FA in the foregut, and about 20% of ingested FA can enter the hindgut [13], suggesting that the gut microbiota can be a functional target site. Our previous study found that dietary FA increased the relative abundances of *Faecalibacterium* and *Faecalicoccus* in the duck cecum, which were reported to produce butyrate and improve intestinal epithelial health [14]. However, the effects of FA on the oxidative stress or gut microbiota in ducks are not clear.

Lipopolysaccharide (LPS) is a major component of the Gram-negative bacteria cell wall, which causes sickness syndromes in animals. One toxicological mechanism for LPS in poultry is to cause oxidative stress [15], and our previous study established an oxidative stress model of Linwu ducks using LPS injections [16]. LPS also disturbed the intestinal microbial homeostasis and damaged the intestinal integrity, which led to intestinal barrier dysfunction and worsened the oxidative stress in birds [17,18]. In the present study, we established an oxidative-stressed Linwu duck model using LPS injections and fed them with a diet supplemented with FA. The aim was to elucidate the effects of FA on the antioxidant stress and gut microbiota imbalance in ducks and explore the mechanism focusing on the gut microbiome and associated metabolites.

## 2. Materials and Methods

### 2.1. Animals and Experiment Design

All animal experiments were conducted according to the guidelines of Laboratory Animal care and use and were approved by the Animal Welfare and Ethics Committee of the Hunan Institute of Animal Husbandry and Veterinary Medicine (approval number: HIAHVM202306). A total of 270 female 28-day-old Linwu ducks obtained from Hunan Shunhua Duck Industrial Development Company (Linwu, China) were involved in the experiment. These ducks were randomly separated into three groups, which were the control group (CON), the LPS-treated group (LPS), and the FA-treated group (LPS-FA). Every 10 ducks were weighted at a time and assigned to one cage as an experimental unit, so that each group had 9 cages of 90 ducks in total. All the ducks were raised in plastic plain netting cages (1.8 m in length, 1.2 m in width, 2 m in height), which were equipped with one feed bucket and a nipple drinking line on the side (3 nipples/10 birds). Birds had no restrictions on actions within the cages and were provided with water and feed ad libitum. Ducks in CON and LPS groups were fed with basal diets which met the nutritional requirements for growing ducks (Table S1) and the ones in the LPS-FA group were fed with basal diets supplemented with 400 mg/kg FA. The feeds were made in New Start Point feed factory (Changsha, China). Intraperitoneal injections of 0.4 mg/kg LPS were administered to ducks in the LPS and LPS-FA groups to trigger oxidative stress, and saline of the same dose was administered to the ones in the CON group on day 27 and 28. The skin surface was sterilized with alcohol before and after injections. FA was purchased from Shanghai Rhawn Chemical Technology Co., Ltd. (Shanghai, China) with ≥99% purity. Escherichia coli O55:B5 LPS was purchased from Sigma (St. Louis, MO, USA). The amounts of dietary FA and injected LPS were determined from our previous experiments [14,16].

### 2.2. Growth Performance

The experimental ducks were weighed based on their cage (replicate) on day 1 and day 29 after 12 h of fasting, recorded as initial body weight (BW) and final BW. Feed consumption for each replicate was collected daily and the total feed intake was calculated. At the end of the experiment, the average daily weight gain (ADG) was calculated by deducting the average initial BW from the average final BW, and the feed-to-gain ratio (F:G) was calculated by dividing the average daily feed intake (ADFI) by the ADG.

### 2.3. Sample Collection

After weighing, one bird from each cage was randomly chosen for sampling (9 ducks in each group and 27 in total). Before blood sampling, the skin surface of the injected location was sterilized with alcohol. Blood was collected from the wing vein with vacuum blood collection tubes, and blood withdrawal was performed with cotton with alcohol. Blood samples were centrifuged at 3000× *g* for 10 min to obtain serum. After blood collection, birds were gently anesthetized by ether and euthanized by cervical dislocation. The sample ducks were then dissected, and the digesta in the cecum were collected separately into 2 mL EP tubes, frozen immediately in liquid nitrogen, and stored at −80 °C for the following analysis.

### 2.4. Oxidative Stress Evaluation

The oxidative stress was evaluated by the levels of oxidative cytokines, antioxidant enzymes, and inflammatory substances in the serum. Malondialdehyde (MDA), glutathione (GSH), glutathione peroxidase (GSH-Px), superoxide dismutase (SOD), catalase (CAT), total antioxidant capacity (T-AOC), nuclear factor κB (NF-κb), tumor necrosis factor α (TNF-α), interferon γ (IFN-γ), interleukin 2 (IL-2), immunoglobulin (Ig) G, and IgA were determined by ELISA kits (Shanghai Jianglai Biotechnology Co., Ltd., Shanghai, China) with a MultiskanTM SkyHigh automated fluorescence instrument (Thermo Fisher Scientific, Waltham, MA, USA) according to the instructions.

### 2.5. Metagenomic Analysis of Cecal Digesta

For each group, six cecal digesta samples (18 samples in total) were randomly chosen for metagenomic and metabolomic analyses. The total genomic DNA was extracted from 1 g of the digesta samples using the E.Z.N.A.^®^ Soil DNA Kit (Omega Bio-tek, Norcross, GA, USA) according to the manufacturer’s instructions. The concentration and purity of the extracted DNA was determined with TBS-380 and the NanoDrop 2000 spectrophotometer (NanoDrop Technologies, Wilmington, DE, USA), respectively. After the DNA extract quality was checked on 1% agarose gel, they were fragmented to an average size of about 400 bp with Covaris M220 (Gene Company Limited, Hongkong, China) for paired-end library construction using NEXTFLEX^®^ Rapid DNA-Seq (Bioo Scientific, Austin, TX, USA). Adapters containing the full complement of sequencing primer hybridization sites were ligated to the blunt end of fragments. Paired-end sequencing was performed on the Illumina Hiseq 3000 platform (Illumina Inc., San Diego, CA, USA) at Majorbio Bio-Pharm Technology Co., Ltd. (Shanghai, China) using HiSeq X Reagent Kits according to the manufacturer’s instructions.

The paired-end reads were trimmed of adaptors and the low-quality reads (length less than 50 bp, with a quality value less than 20, or having N bases) were removed by fastp (https://github.com/OpenGene/fastp, accessed on 26 January 2024). Reads were aligned to the duck (*Anas platyrhynchos*) genome by BWA (http://bio-bwa.sourceforge.net, accessed on 31 January 2024) and any hit associated with the reads and their mated reads were removed. The filtered reads were assembled using MEGAHIT (https://github.com/voutcn/megahit, accessed on 31 January 2024). Qualified contigs with the length being or over 300 bp were selected as the final assembly results, which were used for further analysis. MetaGene (http://github.com/CharlesJB/metagene, accessed on 31 January 2024)was used to predict the open reading frames from the assembled contigs with a length longer than 100 bp. Assembled contigs were then pooled and a non-redundant gene catalog with 90% sequence identity and 90% coverage was constructed using CD-HIT (http://www.bioinformatics.org/cd-hit/, accessed on 31 January 2024). SOAPaligner (http://github.com/ShujiaHuang/SOAPaligner, accessed on 31 January 2024) was used to estimate the abundances of the qualified genes in each sample by comparing with the non-redundant gene catalog with 95% identity.

Bacteria were the dominant domain in the cecal microorganism, so they were the main candidates in the following analysis. Taxonomic and KEGG annotation of the cecal bacteria were performed using DIAMOND (http://github.com/bbuchfink/diamond, accessed on 31 January 2024) with an e-value cutoff of 1e^−5^ referring to the NCBI NR database and the Kyoto Encyclopedia and Genes and Genomes database, respectively. The taxonomy of bacteria was based on the relative number of reads.

### 2.6. Metabolomic Analysis of Cecal Digesta

The metabolites were extracted from 50 mg of defrosted cecal digesta from each sample separately, with 400 μL of 80% methanol buffer and settled at −20 °C. Then, the mixture was treated at 5 °C by a high-throughput tissue crusher (Wonbio-96c, Wanbo Biotechnology Co., Ltd., Shanghai, China) at 50 Hz for 6 min, followed with a vortex for 30 s and ultrasound at 40 kHz for 30 min. After that, the samples were placed at −20 °C for 30 min, allowing the proteins to precipitate. Finally, the samples were centrifugated at 13,000× *g* at 4 °C for 15 min, and the supernatants of each sample were carefully collected for the liquid chromatography–mass spectrometry (LC–MS) analysis.

As a part of the system conditioning and quality control process, a pooled quality control (QC) sample was prepared by mixing equal volumes of all samples. The QC sample was disposed of and tested in the same manner as the tested samples. It helped to represent the whole sample set and monitor the stability of the analysis.

An ultra-performance liquid chromatography (UPLC) system (SCIEX, Milford, MA, USA) and ACQUITY BEH C18 column (100 mm by 2.1 mm, 1.7 μm, Waters, Milford, MA, USA) were used to identify the chromatographic separations. The sample injection volume was set at 2 μL and the flow rate was 0.4 mL/min. The column temperature was maintained at 400 °C and all samples were stored at 4 °C during the period of analysis. The mass spectrometric data were collected using a Thermo UHPLC-Q exactive mass spectrometer equipped with an electrospray ionization (ESI) source operating in either positive or negative ion mode. The detection was carried out over a mass range of 70–1050 m/z.

After UPLC–TOF/MS analyses, the raw data were imported into Progenesis QI 2.3 (Nonlinear Dynamics, Waters, Milford, MA, USA) for peak detection and alignment. The preprocessing results generated a data matrix that consisted of the retention time (RT), mass-to-charge ratio (m/z) values, and peak intensity. Metabolic peaks presented in <20% of any samples or with a relative standard deviation >30% were discarded. The unidentified peaks were removed from the downstream analysis as well. After filtering, the samples in which the metabolite level fell below the lower limit of quantitation were inputted with minimum metabolite values. Each identified metabolite was normalized to log transformed data before entering the following imputation and statistical analysis procedures.

The annotation of metabolites was performed referring to the KEGG database and HMDB based on the exact molecular mass data of samples, MS/MS fragment spectra, and isotope ratio difference. The mass tolerance between the measured molecular mass values and the exact mass was ±10 ppm. For metabolites having MS/MS confirmation, only the ones with MS/MS fragments score above 30 were considered as confidently identified.

### 2.7. Data Analysis

A one-way analysis of variance (ANOVA) and least significant difference (LSD) pos-hoc test was used to determine the differences in oxidative stress-related parameters in serum, as well as the α diversity of gut microbiota among groups, with a *p* value < 0.05 being considered significant. The Wilcoxon rank-sum test and nonmetric multidimensional scaling (NMDS) based on Bray–Curtis dissimilarity matrices were used in the comparison of the microbial communities at the phylum level between groups, with the FDR-adjusted *p* value < 0.05 being considered significant. A linear discriminant analysis effect size (LEfSe) was conducted in the comparison of microbial communities at the genus and species levels, as well as the KEGG pathway between groups, with an LDA score > 2 and a *p* value < 0.05 being considered significant.

A multivariate statistical analysis of metabolites was performed using the ropls R package from Bioconductor (http://bioconductor.org/packages/release/bioc/html/ropls.html, accessed on 31 January 2024) on Majorbio Cloud Platform (https://cloud.majorbio.com). All metabolite variables were scaled to Pareto scaling before following analysis. An orthogonal partial least squares discriminate analysis (OPLS-DA) was used to determine metabolic changes between comparable groups. The model validity was evaluated from model parameters R2 and Q2, which provide information for interpretability and predictability, respectively. Variable importance in the projection (VIP) was calculated in the OPLS-DA model. The *p* values were estimated with an unpaired Student’s t-test on a single dimensional statistical analysis. Statistical significance among groups were determined by a VIP value > 1 and *p* value < 0.05.

## 3. Results

### 3.1. FA Alleviated the Reduction in Growth Performance Induced by LPS

**Figure 1:**
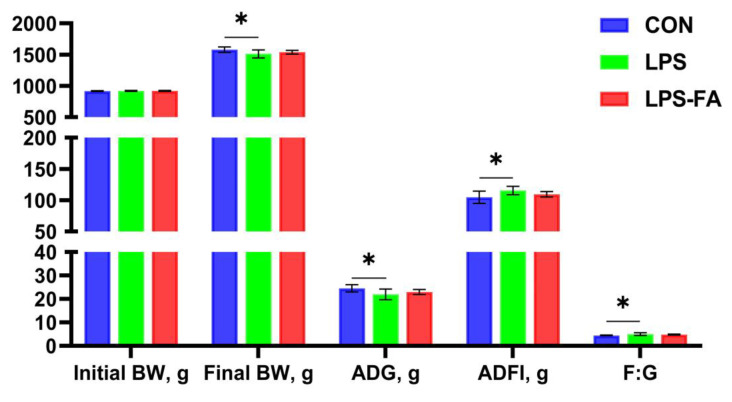
Effects of LPS and FA on the growth performance of Linwu ducks. Data are presented as mean ±SD (*n* = 9) and analyzed by one-way ANOVA. “*” indicates *p* < 0.05. BW, body weight; ADG, average daily weight gain; FI, average daily feed intake; F:G, feed-to-gain ratio.

The parameters of ducks’ growth performances are shown in Figure 1. It shows that LPS significantly reduced the final BW (*p* = 0.048) and the ADG (*p* = 0.039), and increased the ADFI (*p* = 0.038) and F:G (*p* = 0.038) of ducks compared to the CON group. However, 400 mg/kg of dietary FA prevented the effect of LPS on growth performance, and restored the final BW, ADG, ADFI, and F:G to a similar level as CON.

### 3.2. FA Alleviated the Oxidative Stress Induced by LPS

**Figure 2:**
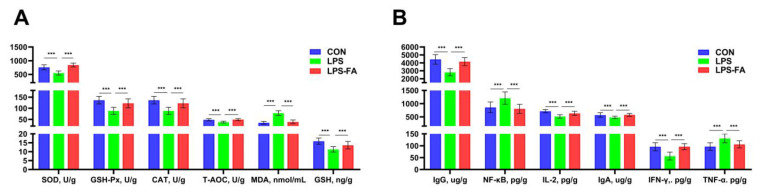
Evaluation of oxidative stress in Linwu ducks subjected to LPS and FA treatments. (**A**) Antioxidant enzymes and oxidative cytokines; (**B**) inflammatory substances. Data are presented as mean ± SD (*n* = 9) and analyzed by one-way ANOVA. “***” indicates *p* ≤ 0.001. SOD, superoxide dismutase; GSH-Px, glutathione peroxidase; CAT, catalase; T-AOC, total antioxidant capacity; MDA, malondialdehyde; GSH, glutathione; Ig, immunoglobulin; NF-κb, nuclear factor κB; IL-2, interleukin 2; IFN-γ, interferon γ; TNF-α, tumor necrosis factor α.

The parameters representing oxidative stress of ducks are shown in Figure 2. Compared to CON, LPS significantly decreased the antioxidant enzyme levels, including GSH-Px (*p* < 0.001), SOD (*p* < 0.001), CAT (*p* < 0.001), and GSH (*p* = 0.001). Furthermore, LPS also increased the concentration of MDA (*p* < 0.001) compared to the CON group. FA alleviated the changes caused by LPS and re-established the measures to normal (similar level as the CON group) (Figure 2A). Similar patterns were found for the inflammatory cytokines (Figure 2B). The NF-κb (*p* < 0.001) and TNF-α (*p* < 0.001) in serum were significantly increased, and IgG (*p* < 0.001), IL-2 (*p* < 0.001), IgA (*p* = 0.009), and IFN-γ (*p* < 0.001) were significantly decreased in the LPS group compared to the CON group. And FA significantly counteracted the effects of LPS on these parameters and restored them to similar levels as the CON group.

### 3.3. Gut Microbial Composition and Function

A total of 1,022,821,466 reads, with 56,823,415 ± 1,543,700 reads per sample, were generated by metagenome sequencing. After quality control and assembly, a total of 4,478,753 contigs were composed, with 248,820 ± 21,402 contigs per sample. The microbial domain of bacteria possessed 99.54% of the cecal metagenome, and these reads were pooled for the following analysis.

**Figure 3:**
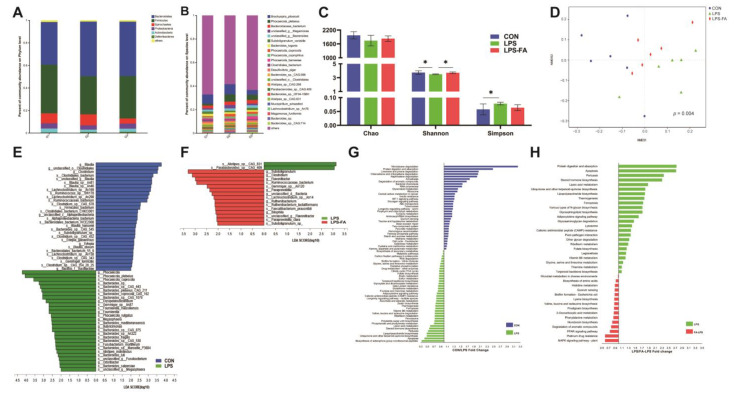
Comparison of bacterial composition and function in ducks’ cecal digesta. (**A**) Gut microbial composition at phylum level. (**B**) Gut microbial composition at species level. (**C**) α-diversity of gut microbiota in ducks. “*” indicates *p* ≤ 0.05. (**D**) Nonmetric multidimensional scaling (NMDS) plots of bacterial communities based on Bray–Curtis distance metric. (**E**) Difference in gut microbiota between CON and LPS. (**F**) Difference in gut microbiota between LPS and LPS-FA. Comparison was conducted by linear discriminant analysis effect size (LEfSe) analysis, with linear discriminant analysis (LDA) score > 2 and *p* < 0.05 being considered significant. (**G**) Comparison of KEGG pathway associated with differential bacteria between CON and LPS. (**H**) Comparison of KEGG pathway associated with differential bacteria between LPS and LPS-FA.

*Bacteroidetes* and *Firmicutes* were two dominant phyla in all three groups, accounting for more than 80% of all replicated metagenomes, followed by *Spirochaetes*, *Proteobacteria*, and *Actinobacteria* (Figure 3A). At the phylum level, the abundance of *Bacteroidetes* was significantly lower in CON compared to LPS (*p* = 0.037), and the abundance of *Firmicutes* was significantly higher in CON compared to LPS (*p* = 0.022). However, FA decreased the abundance of *Bacteroidetes* but increased the abundance of *Firmicutes* compared to LPS. At the species level, the dominant bacteria of the three groups were *Brachyspira pilosicoli*, followed by *Phocaeicola plebeius* and *Bacteroidaceae bacterium*, and the species with a relative abundance larger than 1% are shown in Figure 3B. The comparison of α diversity is shown in Figure 3C; the Shannon index was significantly lower (*p* = 0.037) but the Simpson index (*p* = 0.041) was significantly higher in LPS compared to CON. On the other hand, the Shannon index in LPS-FA was significantly higher than LPS. No differences were observed in the Chao indexes among all three groups (*p* = 0.108).

The NMDS with an analysis of similarities (ANOSIM) showed a separation of bacteria composition at the species level between CON and LPS, but no difference was detected between LPS and LPS-FA (*p* = 0.004; Figure 3D). With the LEfSe test between CON and LPS, 30 taxa were significantly enriched in CON, including six genera and 24 species, and 28 taxa were significantly enriched in LPS, including six genera and 22 species (linear discriminant analysis [LDA] > 2 and *p* < 0.05; Figure 3E). In the LEfSe test between LPS and LPS-FA, two taxa were significantly enriched in the LPS, and 15 taxa were significantly enriched in the LPS-FA, including seven genera and eight species (LDA > 2 and *p* < 0.05; Figure 3F).

Furthermore, we identified the potential functions of the differential bacterial taxa by deciphering the metagenomic information. It was shown that 34 and 37 KEGG pathways were significantly enriched in the CON and LPS groups, respectively (LDA > 2 and *p* < 0.05; Figure 3G), and 24 and 15 KEGG pathways were significantly enriched in the LPS and LPS-FA groups, respectively (LDA > 2 and *p* < 0.05; Figure 3H).

### 3.4. Cecal Metabolomic Profiles in Ducks

**Figure 4:**
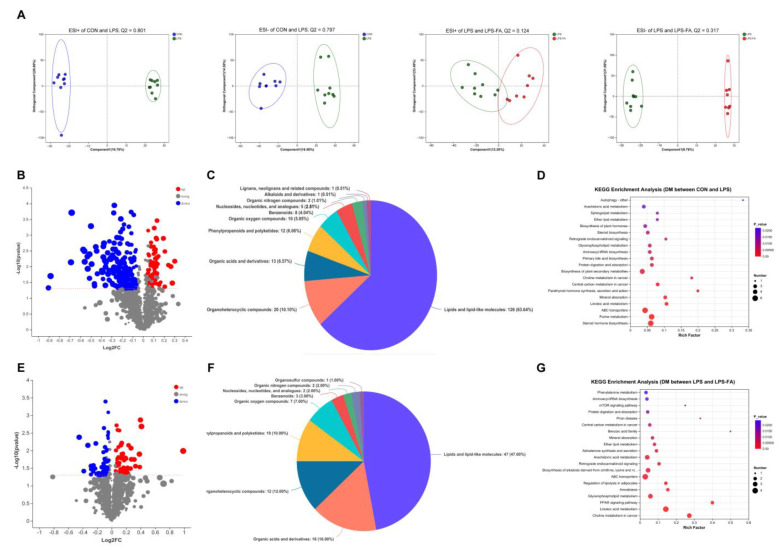
Comparison of metabolite composition in ducks’ cecal digesta. (**A**) OPLS-DA score plots of ESI+ and ESI−. (**B**) Volcano plot of the different metabolites (DMs) between CON and LPS. (**C**) Classification of the DMs between CON and LPS at the superclass level against the HMDB. (**D**) Enrichment analysis of KEGG pathway based on the DM between CON and LPS. (**E**) Volcano plot of the DMs between LPS and LPS-FA. (**F**) Classification of the DMs between LPS and LPS-FA at the superclass level against the HMDB. (**G**) Enrichment analysis of KEGG pathway based on the DMs between LPS and LPS-FA.

We further looked into the metabolome in the cecal digesta of the experimental ducks via UPLC–MS methods. The orthogonal partial least squares discrimination analysis (OPLS-DA) with the unpaired Student’s *t*-test showed that the cecal metabolites of CON and LPS, as well as LPS and LPS-FA, were clearly discriminated (Figure 4A). A total of 254 differential metabolites (DMs) were found between CON and LPS, of which 175 DMs were up-regulated and 79 DMs were down-regulated in LPS compared to CON (Figure 4B, Table S2). By referring to the human metabolome database (HMDB) at the superclass level, 63.64% DMs were lipids and lipid-like molecules, followed by 10.10% organoheterocyclic compounds and 6.57% organic acids and derivatives (Figure 4C). The KEGG pathway analysis revealed that 35 pathways were significantly enriched, such as “steroid hormone biosynthesis”, “protein digestion and absorption”, “ABC transporters”, “glutathione metabolism”, “choline metabolism in cancer”, and others. (*p* ≤ 0.05; Figure 4D).

Regarding the comparison between LPS and LPS-FA, a total of 125 DMs were identified, of which 70 DMs were up-regulated and 55 DMs were down-regulated in LPS-FA compared to LPS (Figure 4E, Table S3). By the HMDB classification at the superclass level, 47.00% DMs were lipids and lipid-like molecules (47.00%), followed by organic acids and derivates (16.00%) and organoheterocyclic compounds (12.00%) (Figure 4F). The KEGG pathway analysis revealed significant enrichments of 39 pathways, such as “phenylalanine metabolism”, “peroxisome proliferator activated receptor (PPAR) signaling pathway”, “protein digestion and absorption”, “amoebiasis”, “ABC transporters”, and others, (*p* ≤ 0.05, Figure 4G).

### 3.5. Integrative Analysis of Metagenome and Metabolome in Gut Microbiota

**Figure 5:**
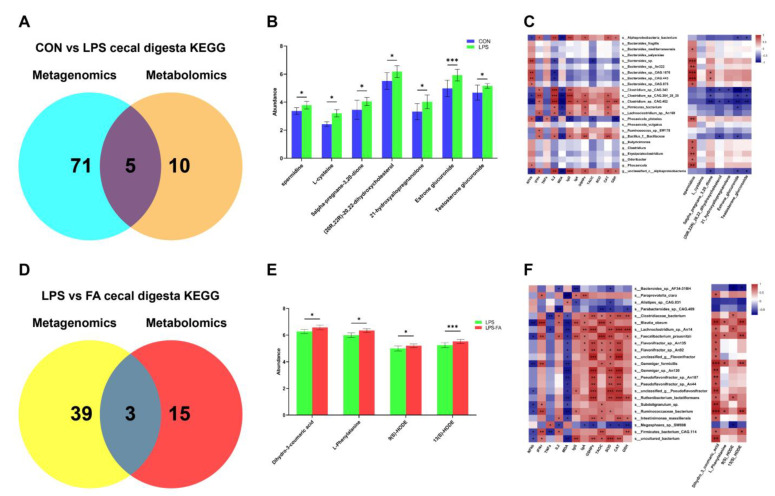
Integrated analysis of metagenome and metabolome in cecal digesta of ducks. (**A**) Venn diagram of KEGG pathways enriched from differential bacteria and differential metabolites in cecal digesta of ducks between CON and LPS. (**B**) Differential metabolites involved in pathways of glutathione metabolism and steroid hormone biosynthesis between CON and LPS. (**C**) Correlation of differential bacteria with metabolites and antioxidant-related cytokines between CON and LPS. (**D**) Venn diagram of KEGG pathways enriched from differential bacteria and differential metabolites in cecal digesta of ducks between CON and LPS. (**E**) Differential metabolites involved in pathways of phenylalanine metabolism and PPAR signaling pathway between CON and LPS. (**F**) Correlation of differential bacteria with metabolites and antioxidant-related cytokines between LPS and LPS-FA.

In the metagenome and metabolome of gut bacteria between the CON and LPS, five KEGG pathways were commonly identified, which were ABC transporters, central carbon metabolism in cancer, protein digestion and absorption, glutathione metabolism, and steroid hormone biosynthesis (Figure 5A). Both omics suggested that pathways of glutathione metabolism and steroid hormone biosynthesis were up-regulated in LPS compared to CON. DMs involved in the pathway of glutathione metabolism included spermidine (*p* = 0.034) and L-cysteine (*p* = 0.013), which were significantly increased in LPS compared to CON. And DMs related to the steroid hormone biosynthesis included 5α-pregnane-3,20-dione (*p* = 0.028), (20R,22R)-20,22-dihydroxycholesterol (*p* = 0.013), estrone glucuronide (*p* = 0.001), 21-hydroxyallopregnanolone (*p* = 0.015), and testosterone glucuronide (*p* = 0.022), which were significantly increased in LPS compared to CON (Figure 5B). Furthermore, correlations among the differential bacteria, DMs, and oxidative stress-related cytokines between CON and LPS are visualized (Figure 5C). *S_Clostridium*_sp._*CAG.452*, *s_Clostridium*_sp._*CAG.353_28_25*, and *g_Bacillus* were positively associated with GSH-Px, CAT, IFN-γ, IL-2, IgG, and other antioxidant substances and enzymes, and negatively associated with MDA and NF-κb (*p* < 0.05). These taxa were also negatively associated with estrone glucuronide, testosterone glucuronide, and other steroid hormone biosynthesis-related DMs (*p* < 0.05).

When comparing the LPS and LPS-FA groups, three pathways were commonly found, which were protein digestion and absorption, phenylalanine metabolism, and the PPAR signaling pathway (Figure 5D). Both omics suggested that phenylalanine metabolism and the PPAR signaling pathway were up-regulated in LPS-FA compared to LPS. With the DMs between LPS and LPS-FA, dihydro-3-coumaric acid (*p* = 0.030) and L-Phenylalanine (*p* = 0.031) were associated with phenylalanine metabolism, and 9(S)-HODE (*p* = 0.010) and 13(S)-HODE (*p* < 0.001) were associated with the PPAR signaling pathway, which were all significantly increased in LPS-FA compared to LPS (Figure 5E). The correlation analysis suggested that *s_Balutia_obeum*, *s_Fecalibacterium_prausnitzii*, and *s_Ruminococcaceae_bacterium* were positively associated with the majority of the antioxidant enzymes and anti-inflammatory cytokines, and negatively associated with MDA and NF-κb (*p* < 0.05). Meanwhile, these taxa were positively associated with dihydro-3-coumaric acid, L-phenylalanine, and 13(S)-HODE (*p* < 0.05).

## 4. Discussion

Oxidative stress is a common problem in the modern poultry farming industry. It is induced by factors such as excessive density, heat, malnutrition, and pathogens [2]. LPS is a mycotoxin that induces oxidative stress in birds. We successfully established oxidative-stressed Linwu ducks by injections of LPS [16]. In the present study, we aimed to trigger oxidative stress in Linwu ducks with LPS injections and investigate the effects of FA on the growth performance, oxidative status, and gut microbiota composition of the ducks. Results showed that LPS damaged growth performance by reducing the final BW and ADG and increasing the ADFI and F:G in 28-day-old to 56-day-old ducks. Jeopardized growth performance could be induced by severe oxidative stress caused by LPS. A similar result was found by Yang et al. [19], where LPS reduced the BW and ADG of broilers. However, a dietary supplement of FA increased the BW and ADG, and decreased the ADFI and F:G to a similar level as the CON group. This result was in line with a previous study on geese, where dietary FA increased the ADG and reduced the F:G compared to ones injected with LPS [20]. It is suggested that 400 mg/kg of FA supplemented in the diet of ducks could alleviate the damage in growth performance induced by LPS.

Furthermore, we evaluated the oxidative stress in ducks by investigating the antioxidant enzymes and stress-related cytokines. SOD, GSH-Px, and CAT are water-soluble antioxidant enzymes that serve as the initial level of the antioxidant defense system in birds [2]. GSH is essential for eliminating hydroperoxide and scavenging free radicals [21]. These cytokines represent the antioxidant capacity of the organism. MDA, as the product of lipid peroxidation, is usually measured as an indicator of oxidative stress severity in the body [7]. Moreover, oxidative stress is associated with immune responses in birds, as exposure to oxidative stress causes immuno-pathological damage [22], so that inflammatory cytokines such as NF-κb, TNF-α, and others can reflect the oxidative stress in birds as well. In the present study, ducks in the LPS group had lower levels of SOD, GSH-Px, CAT, GSH, and T-AOC, and higher levels of GSH compared to CON. Meanwhile, LPS reduced the concentration of IgA, IgG, IL2, and IFN-γ, but increased the concentration of NF-κb and TNF-α. These results indicate that severe oxidative stress and immune response in ducks were induced by LPS. Similar results were reported previously in broilers, hens, and piglets [23,24,25]. Intriguingly, dietary FA prevented the influence of LPS on the oxidative stress and inflammation-related cytokines, showing a strong antioxidant effect. This finding was supported by a previous study in piglets, demonstrating that dietary FA increased antioxidant enzyme levels and decreased the MDA concentration in the serum and liver of weaned piglets [26]. Shu et al. [27] also reported that dietary FA reduced SOD, T-AOC, and GSH levels in the liver of LPS-treated broilers.

In order to understand the effect of FA on the gut microbiota of Linwu ducks, we conducted metagenomic and metabolomic analyses of the cecal digesta. Previous studies stated that LPS disturbed the microbial homeostasis by changing the composition and diversity of gut microbiota [28,29]. In our study, LPS reduced the diversity and abundance, but increased the uniformity of the gut microbiota in ducks. And FA could alleviate the effects of LPS by increasing microbial diversity. LPS increased the abundance of *Bacteroidetes* and decreased the abundance of *Firmicutes*. *Bacteroidetes* and *Firmicutes*, two dominant bacteria phyla in ducks. *Firmicutes* was associated with the energy harvesting efficiency in birds [30], and the decreased *Firmicutes/Bacteroidetes* ratio induced by LPS suggested disorders in gut microbiota [31]. However, these changes were relieved by FA, as the abundance of *Bacteroidetes* and *Firmicutes* were restored, and the α diversity was recovered in LPS-FA compared to LPS. A similar result was reported by Hu et al. [32], showing that FA modulated the gut microbiota composition of LPS-challenged piglets by increasing the relative abundance of *Firmicutes* and decreasing the relative abundance of *Bacteroidetes.* The LEfSe analysis further determined the differential bacteria taxa among groups. From the enriched bacteria in the LPS group, *g_Butyricimonas* was reported to correlate with inflammatory and oxidative parameters in alcohol-treated mice [33], and *g_Odoribacter* was considered as an atypical pathogen which was involved in the inflammatory process as well [34]. The majority of the differential bacteria enriched in LPS-FA were short-chain fatty acids producers, which were beneficial to the immune and antioxidant status of the host [35,36,37]. Additionally, functions of the differential bacteria were explored, revealing that 34 and 37 KEGG pathways were enriched in CON and LPS, respectively, and 24 and 15 KEGG pathways were enriched in LPS and LPS-FA, respectively. These differential bacteria and functions served as potential biomarkers for LPS- and FA-treated ducks.

Changes in the intestinal bacteria composition resulted in alternations in microbial metabolites, which influenced the physiological reactions of the organism [38]. Here, we analyzed the metabolites of the cecal digesta using a non-target UPLC–MS analysis. A total of 254 DMs were identified between CON and LPS, and 125 DMs were identified between LPS and LPS-FA. These DMs were mainly enriched in lipid metabolism and amino acid metabolism. Gut microbiota created molecules through biosynthesis and the modification of host substrates. These molecules, including lipids and amino acids, had a profound influence on host health [39]. LPS-induced oxidative stress caused growth inhibition in birds, which was associated with an alternation of nutrient metabolism. Under stress conditions, birds needed extra nutrients and energy to synthesis immune and antioxidant effector molecules, which enhanced the catabolism of protein and fat instead of anabolism [40]. A previous study on broilers using a gut microbiomics and metabolomics analysis stated that stress induced by LPS and the protection effect of lauric acid were associated with lipid metabolism and amino acid metabolism, which is in line with our finding [41]. Consequently, these metabolites and metabolic pathways served as potential biomarkers in LPS-induced oxidative stress and the antioxidant effect of FA in ducks.

The integrated analysis of gut microbial metagenomics and metabolomics data was further conducted to screen the critical biomarkers and mechanisms involved in the effects of LPS and FA on ducks. In our study, KEGG pathways of glutathione metabolism and steroid hormone biosynthesis were found to be up-regulated in LPS compared to CON under both omics analyses. With the Spearman correlation analysis, (20R,22R)-20,22-dihydroxycholesterol, estrone glucuronide, and testosterone glucuronide, which were metabolites involved in steroid hormone biosynthesis, were found to be negatively associated with *s_Clostridium_*sp.*CAG.452*, *s_Clostridium_*sp.*_CAG.354_28_25*, and *g_Bacillus*. (20R,22R)-20,22-dihydroxycholesterol was the precursor of corticosterone, which is the primary glucocorticoid in birds [42]. Corticosterone is treated as an indicator of stress in birds, as its expression is enhanced to overcome oxidative and immunological stress under the stimulation of LPS [43]. Estrone glucuronide, as glucuronic acidified estrone, can be modified by bacterial β-glucuronidase in the intestine, and the estrogen can be reabsorbed into the enterohepatic circulation [44]. The circulating estrogen is essential for the estrogen balance, which interferes with the lipid metabolism and fatty liver development in birds [45]. Testosterone and its glucuronic acidified form can be modified by gut microbes through the degradation pathway [46]. It was possible that LPS disturbed the gut microbiota composition in ducks and disturbed the microbial function of estrogen circulation and testosterone modification, causing the accumulations in estrone glucuronide and testosterone glucuronide. Previous studies stated that *s_Clostridium_*sp.*CAG.452*, *s_Clostridium_*sp.*_CAG.354_28_25* (both belonged to the genera of *Clostridium*), and *g_Bacillus* might possess the function of steroid hormone biosynthesis, and that species of *Clostridium*- and *Bacillus-*encoded hydroxysteroid dehydrogenases participated in microbial bile acid metabolism and cortisol metabolism [47,48]. These activities supported our findings of the potential correlations between the microbes and steroid hormone-related metabolites. Furthermore, we found that *s_Clostridium_*sp.*CAG.452*, *s_Clostridium_*sp._*CAG.354_28_25*, and *g_Bacillus* showed positive correlations with the antioxidant capacity of ducks. Previous studies suggested that probiotics in *Clostridium* and *Bacillus*, such as *Clostridium butyricum*, *Bacillus subtilis, Bacillus licheniformis*, and *Bacillus coagulans*, improved the antioxidant capacity of birds [49,50,51], which is in line with our findings, stating that these microbes were associated with the antioxidant-associated cytokines. In all, we concluded that *s_Clostridium_*sp.*CAG.452*, *s_Clostridium*_sp._*CAG.354_28_25*, and *g_Bacillus* could be the biomarkers that identified differences between CON and LPS, and their interactions with steroid hormone biosynthesis-related metabolites, such as (20R,22R)-20,22-dihydroxycholesterol, estrone glucuronide, and testosterone glucuronide, might contribute to the oxidative stress induced by LPS.

In regard to the comparison between LPS and LPS-FA, KEGG pathways of phenylalanine metabolism and the PPAR signaling pathway were found to be up-regulated in LPS-FA compared to LPS in both omics analyses. In the correlation analysis, *s_Blautia_obeum*, *s_Faecallbacterium_prausnitzii*, *s_Gemmiger_formicilis,* and *s_Ruminococcaceae_bacterium* were correlated with phenylalanine metabolism-associated metabolites (dihydro-3-coumaric acid and L-phenylalanine) and the PPAR signaling pathway-associated metabolite (13(S) HODE). Dihydro-3-coumaric acid is a phenolic acid metabolized from phenylalanine, which possesses antioxidant activity because of its chemical structure [52]. A previous study in laying hens reported that L-phenylalanine was positively correlated with T-AOC and SOD in plasma, suggesting L-phenylalanine is a biomarker for antioxidant capacity in birds. 13(S)-HODE is a ligand for PPARγ, which shows an agonist effect on the activation of PPARγ [53,54]. PPARγ and its agonists can inhibit the NF-κb pathway to regulate antioxidant response elements and inflammatory factors [55]. In addition, *s_Blautia_obeum*, *s_Faecalibacterium_prausnitzii*, *s_Gemmiger_formicilis*, and *s_Ruminococcaceae_bacterium* were found also positively correlated with antioxidant cytokines and negatively correlated with MDA and NF-κb in our study, suggesting their positive correlations with the antioxidant capacity of ducks. Similarly, *s_Blautia_obeum* belongs to the genera of *Blautia*, which was reported to be positively correlated to the antioxidant capacity of the gut microbiota and host in a human trial [56]. *S_Faecalibacterium_prausnitzii* produces energy substances, as well as anti-inflammatory metabolites [57], and *s_Ruminococcaceae_bacterium*, as a member of the family *Ruminococcaceae*, has the potential to generate butyrate and other short-chain fatty acids [58]. They were reported as probiotics that maintained the intestinal health and contributed to the enhanced antioxidant capacity of birds [59,60]. Hou et al. [61] reported that the abundance of *s_Gemmiger_formicilis* was increased in dextran sulfate sodium-induced mice after treatment with dietary taxifolin, which was negatively associated with the NF-κb signaling pathway. With all the above, we concluded that *s_Blautia_obeum*, *s_Faecalibacterium_prausnitzii*, *s_Gemmiger_formicilis*, and *s_Ruminococcaceae_bacterium* could be the biomarkers that identified differences between LPS and LPS-FA, and their interactions with phenylalanine metabolism and PPAR signaling pathway-associated metabolites, such as dihydro-3-coumaric acid, L-phenylalanine, and 13(S)-HODE, could be a mechanism for the antioxidant effect of FA.

## 5. Conclusions

This study determined the beneficial effects of FA on LPS-induced oxidative stress in Linwu ducks, including recovering growth performance, relieving oxidative stress, and regulating gut microbiota composition. In the omics analysis, we found 58 bacterial taxa and 254 metabolites being discriminatory between the CON and LPS groups, and 17 bacterial taxa and 125 metabolites being discriminatory between the LPS and LPS-FA groups. The integrated analysis showed that *s_Clostridium_sp.CAG.452*, *s_Clostridium_sp._CAG.354_28_25*, and *g_Bacillus* could be the biomarkers in LPS-induced oxidative stress in ducks, which interacted with (20R,22R)-20,22-dihydroxycholesterol, estrone glucuronide, and testosterone glucuronide, and regulated steroid hormone biosynthesis. In addition, *s_Blautia_obeum*, *s_Faecalibacterium_prausnitzii*, *s_Gemmiger_formicilis*, and *s_Ruminococcaceae_bacterium* could be the biomarkers in the antioxidant effect of FA, which interacted with dihydro-3-coumaric acid, L-phenylalanine, and 13(S)-HODE, and regulated the phenylalanine metabolism and PPAR signaling pathway. These findings provided new insights for LPS-induced oxidative stress in ducks and explored the mechanism of the antioxidant effect of FA. Furthermore, this study offered evidence for FA as a new antioxidant in poultry feed. Future studies are needed to validate the function of the identified biomarkers to establish a solid relationship between FA and the antioxidant capacity of the host.

## Data Availability

The raw data of this project have been deposited in the National Center for Biotechnology Information (NCBI) database (access number: PRJNA723283). All data are available from authors upon reasonable request.

## Supplementary Materials

The following supporting information can be downloaded at: https://www.mdpi.com/article/10.3390/antiox13101190/s1. Table S1: Ingredients and nutrient composition of the basal diet (dry matter basis, %). Table S2: Different metabolites between the CON and LPS groups. Table S3: Different metabolites between the LPS and FA-LPS groups.
