# Supplementary material for: Effects of Ferulic Acid on Lipopolysaccharide-Induced Oxidative Stress and Gut Microbiota Imbalance in Linwu Ducks
Source: Antioxidants (Basel). 2024 Sep 30;13(10):1190. doi: 10.3390/antiox13101190 (PMC11504935; doi:10.3390/antiox13101190)
Supplement: Supplementary file 1 [file antioxidants-13-01190-s001.zip › antioxidants-3184098-supplementary/Table S1.pdf]

Table S1. Ingredients and nutrient composition of the basal diet (dry matter basis, %).

**Ingredients**		**Nutreient Levels** [1]	
Corn	50.68	Metablic enegery, MJ/kg	11.30
Soybean meal	24.50	Dry matter (DM)	87.3
Flour	10.00	Crude protein (CP)	17
Wheat middings	7.00	Calcium (Ca)	0.90
$CaHPO_4$	1.30	Total phosphorus (TP)	0.56
Salt	0.30	Available phosphorus (AP)	0.35
L-Lysine H_2SO_4	0.27	Salt	0.33
DL-Methionine	0.12	Lysine	0.9
Limestone	1.20	Methionine	0.4
Bentonite	3.63	Methionine and cystine	0.789
Premix [2]	1.00	Isoleucinese	0.732
		Threonine	0.6
		Tryptophane	0.264

[1] The nutrient levels were calculated values; [2] The premix provided the following nutrients per kg diet: vitamin A 12,000 IU; vitamin D_3 2,500 IU; vitamin E 20 mg; vitamin K_3 3 mg; vitamin B_1 3 mg; vitamin B2 8 mg; vitamin B6 7 mg; vitamin B12 0.03 mg; *D*-pantothenic acid 20 mg, nicotinic acid 50 mg, biotin 0.1 mg, folic acid 1.5 mg, Cu (as copper sulfate) 9 mg, Zn (as zinc sulfate) 110 mg, Fe (as ferrous sulfate) 100 mg, Mn (as manganese sulfate) 100 mg, Se (as sodium selenite) 0.16 mg, I (as potassium iodide) 0.6 mg.
